# Bitter Compounds in Medicinal Food Plants Based on Traditional Chinese Medicine: Analysis and Regulation Strategies from Chemical Structure to Perception Mechanisms

**DOI:** 10.3390/molecules31122192

**Published:** 2026-06-22

**Authors:** Yuanyuan Li, Nana Feng, Di Yang, Qian Zhang, Xinyan Zhao, Xing Yang, Qingya Yu, Zhaotong Cong, Tingting Kuang, Ce Tang, Yi Zhang

**Affiliations:** 1 Chinese Medicine Germplasm Resources Innovation and Effective Uses Key Laboratory of Sichuan Province, School of Pharmacy, Chengdu University of Traditional Chinese Medicine, Chengdu 611137, China; liyuanyuan2@stu.cdutcm.edu.cn (Y.L.);; 2 School of Ethnic Medicine, Chengdu University of Traditional Chinese Medicine, Chengdu 611137, China; 3 Innovative Institute of Chinese Medicine and Pharmacy, Chengdu University of Traditional Chinese Medicine, Chengdu 611137, China

**Keywords:** bitter compounds, medicinal food plants, TAS2R, bitterness regulation, phytochemicals, bioactivity, sensory modulation

## Abstract

Bitter phytochemicals, including alkaloids, terpenoids, and bitter glycosides, are abundant in medicinal food plants and exhibit well-documented anti-inflammatory, hypoglycemic, and other bioactivities relevant to human health. However, the inherent bitterness of these compounds presents a significant sensory barrier to patient compliance and limits their application as functional food ingredients. This review provides a comprehensive and interdisciplinary synthesis of current knowledge on bitter compounds in medicinal food plants, integrating perspectives from phytochemistry, molecular pharmacology, and sensory science. We summarize the major chemical classes of bitter phytochemicals, critically evaluate methods for their isolation and identification—from classical sensory-guided fractionation to modern computational approaches such as molecular docking and metabolomics—and analyze three principal strategies for bitterness regulation: physical removal, biological transformation, and sensory modulation (including molecular inclusion and TAS2R receptor blocking). We also briefly touch upon the extraoral expression of TAS2Rs and there suggested links to local immune responses and metabolic regulation, noting that this may be relevant to the concept of “taste–bioactivity homology.” The review further highlights ongoing challenges, such as the identification of unknown bitter compounds and the lack of standardized sensory evaluation systems, and outlines possible directions for improving bitterness analysis and regulation in medicinal food plants.

## 1. Introduction

**Figure 1:**
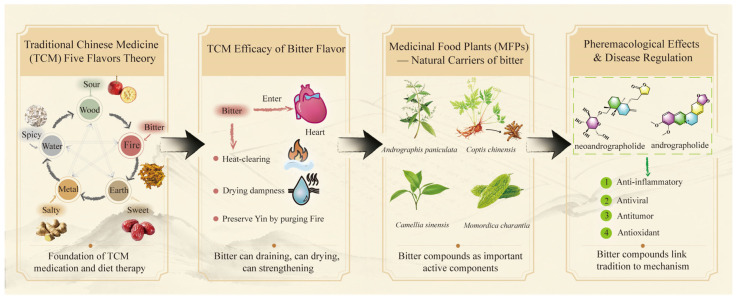
Theoretical framework of bitter compounds in medicinal food plants: from TCM five flavors theory to modern pharmacological applications.

As early as over two millennia ago, the medical classic Huangdi’s Internal Classic (Suwen: Zhizhenyao Dalun) established bitterness within the pivotal framework of yin-yang classification and herbal property theory, stating that “acrid and sweet flavors disperse and pertain to yang, while sour and bitter flavors induce purgation and pertain to yin.” [1]. In traditional Chinese medicine theory, the “Five Flavors” (sour, bitter, sweet, salty and spicy) are the core compounds of the theory of medicinal properties. Bitter taste is “Yin in nature, capable of draining, drying and strengthening”, that is, it has the functions of heat-clearing and purging fire, descending counterflow and promoting purging, drying dampness and strengthening Yin [2]. In modern pharmacological research, the characteristics of bitterness, namely ‘draining, drying, and firming’, are primarily manifested in the pharmacological effects of bitter traditional Chinese medicines, which can exert antipyretic, antibacterial, anti-inflammatory, and antiviral actions. According to statistical data, among the 2711 kinds of Chinese medicinal materials included in the Pharmacopoeia of the People’s Republic of China (2020 Edition), there are 1015 kinds of bitter-tasting drugs, accounting for 37.44%, which occupy a prominent proportion among various flavors [3]. Behind this figure lies the projection of the long-term deep reliance on the medicinal effects of bitter taste in traditional Chinese medicine clinical practice (Figure 1).

Medicinal food plants (MFPs) serve as natural carriers of this bitter taste effect. In this review, the term “medicinal food plants (MFPs)” refers specifically to plant species that are officially recognized both as food and as traditional Chinese medicine materials, and does not indiscriminately include all herbal drugs or functional food ingredients. With the rising global awareness of health and the increasing diversification of dietary culture, medicinal and food plants that possess both edible and medicinal values have attracted growing attention worldwide [4,5,6,7]. Many commonly used medicinal food plants, including dandelion, chicory, andrographis paniculata and bitter melon, are rich in secondary metabolites such as alkaloids, terpenoids, polyphenols and bitter aglycones. These compounds not only constitute the material basis of bitter taste, but also serve as key active substances to exert multiple health benefits, including anti-inflammatory, hypoglycemic, antibacterial and antioxidant effects [8,9,10]. This type of plant perfectly embodies the concept of “taste and effect sharing the same origin”, but due to its prominent bitter taste, it often restricts public acceptance and the standardized application of MFPs in both dietary and clinical contexts.

Faced with this challenge, ancient Chinese physicians long ago devised a practical solution: taste modification through rational herbal compatibility. Under the formulation principle of monarch, minister, assistant and guide, sweet and mild ingredients such as licorice, jujube and honey are commonly applied to moderate the intense bitterness of other herbs. This approach can protect the stomach and regulate qi, without compromising the therapeutic efficacy of principal medicines. This approach of rational taste correction based on experience and compatibility was an early prototype of taste regulation technology and is in line with today’s goal of improving the compliance of bitter preparations in terms of concept [11]. However, in the practice of modern ethnopharmacology, a strong bitter taste remains a significant obstacle restricting patients’ long-term use of herbal preparations derived from MFPs. Especially for the conditioning of chronic diseases that require long-term medication, the tension between improving palatability and preserving efficacy has become a core challenge in the development of medicinal food plant products.

The breakthrough progress in modern bitter taste perception research originated from the discovery of type 2 taste receptors (TAS2Rs). In humans, TAS2Rs represent a subfamily of G-protein-coupled receptors with 25 identified members. The expression of TAS2Rs in taste buds is an important receptor for the perception of bitterness, mediating bitter taste perception in taste cells through the activation of the Gαgust-PLCβ2-IP3-Ca^2+^ pathway [12]. TAS2Rs are also expressed in the oral peripheral tissues of the respiratory, digestive, and reproductive systems, participating in the regulation of physiological functions such as immunity and metabolism [13]. However, the bitter compounds in medicinal food plants have complex and variable structures, with significant differences in content, and coexist with other active substances in the same substrate, which greatly increases the difficulty of separation, identification and targeted evaluation. Although the traditional sensory-guided separation method is direct and reliable, it is time-consuming, labor-intensive, has a low throughput, is prone to losing trace components, disrupts the multi-component synergy effect, and cannot directly locate the interaction of specific TAS2R subtypes. Metabolomics can correlate non-targeted spectra with sensory data to high-throughput identify candidate bitter molecules. However, co-effusions in complex chemical contexts, matrix effects, and database limitations can easily lead to false positives or structures that are difficult to annotate, and can only establish correlations but cannot directly prove the receptor binding mechanism. Molecular docking can rapidly predict the binding potential of compounds with TAS2R, with low cost and high throughput. However, the prediction accuracy depends on the quality of the receptor model, and usually ignores the interaction between bioavailability and components in the substrate. When applied alone in complex plant systems, the uncertainty is relatively high. Although emerging high-throughput methods such as computer virtual screening and metabolomics can complement each other [14,15], their accuracy and applicability in complex systems still need to be further verified. In addition, there are still vast uncharted territories regarding the selectivity of TAS2Rs subtypes, multi-site synergistic activation, and the structure-activity patterns of extraoral effects.

In the face of all the above-mentioned challenges, this article aims to construct a systematic knowledge framework that integrates traditional Chinese medicine theory, modern molecular mechanisms, chemical structures and sensory regulation. This article starts with the natural sources and chemical types of bitter compounds in medicinal food plants, then integrates the methodological system of their separation, identification and sensory-activity evaluation, subsequently explains the perception and functional characteristics mediated by TAS2Rs, and finally reviews the advantages and disadvantages as well as applicable scenarios of various bitter taste removal and flavor masking technologies, and looks forward to the future development direction. This integrated analysis seeks to underpin the rational development of medicinal food plants by reconciling traditional therapeutic experience with contemporary research evidence. Optimizing the sensory performance of these products while preserving and potentiating their bioactivities will enable their broader adoption in modern daily healthcare.

## 2. Sources and Classification of Bitter Compounds

**Figure 2:**
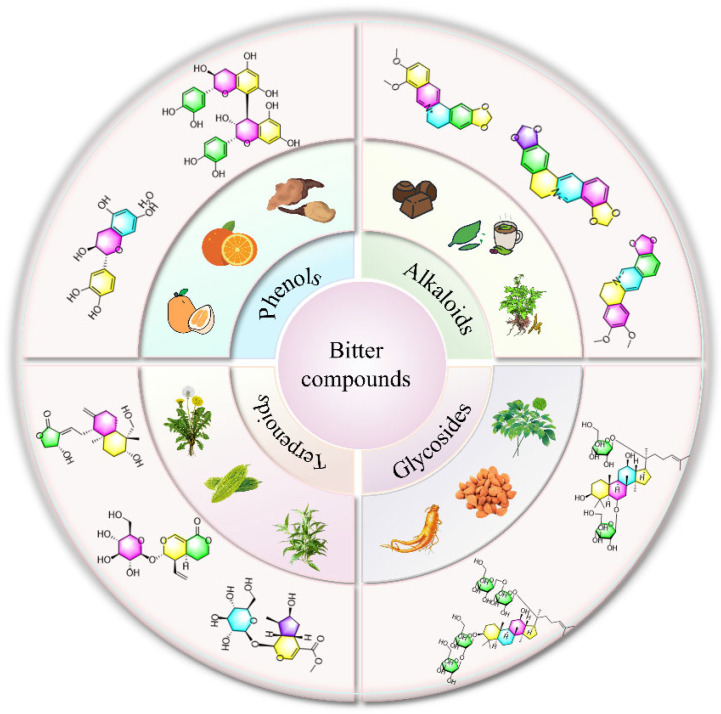
Classification and structural examples of the main bitter compounds.

Bitterness, as a fundamental taste sensation, is widely distributed in MFPs and serves as a key indicator of their pharmacological properties and therapeutic characteristics. Within the theoretical framework of traditional Chinese medicine, bitterness is recognized as one of the five fundamental flavors and is clearly associated with specific medicinal properties. The traditional saying that good medicine tastes bitter but benefits health reflects a long-recognized link between bitterness and therapeutic efficacy. These dual sensory and functional attributes are primarily determined by specific chemical constituents in plants, referred to as bitter compounds. This chapter will be developed in sequence from three interrelated dimensions: source distribution, traditional medicinal uses, and chemical structure classification. It will also use TAS2Rs as molecular links to connect traditional efficacy cognition with modern biochemical mechanisms. Bitter compounds exhibit diverse chemical structures, including alkaloids, glycosides, phenols, terpenoids, peptides, and amino acids (Figure 2). Elucidation of these constituents represents a crucial chemical entry point for understanding the homology of medicine and the food value of medicinal food plants.

### 2.1. Sources of Bitter Compounds

Bitterness, one of the five basic tastes, is widely present in nature [16]. From an evolutionary standpoint, bitter taste perception is considered a protective mechanism that helps mammals identify and avoid consuming potentially toxic plant metabolites [17]. However, bitterness is not always a definitive indicator of toxicity. An increasing number of studies have demonstrated that bitter compounds possess a range of other physiological functions [18]. In the long history of traditional Chinese medicine, the medicinal value of bitter compounds has been systematically recognized and applied, forming a theoretical framework centered on the idea that “bitterness can purge, dry, and firm.” This concept is widely used in the treatment of conditions such as clearing heat, purging fire, drying dampness, detoxifying, and promoting defecation and diarrhea.

Bitter compounds are widely distributed across the plant kingdom, with particularly high concentrations in families such as Compositae (e.g., *Artemisia annua* L.), Rutaceae (e.g., *Phellodendron amurense* Rupr.), Liliaceae (e.g., *Fritillaria cirrhosa* D. Don), Leguminosae (e.g., *Sophora flavescens* Aiton), Lamiaceae (e.g., *Scutellaria baicalensis* Georgi), Apiaceae (e.g., *Angelica sinensis* (Oliv.) Diels), and Araliaceae (e.g., *Panax notoginseng* (Burkill) F. H. Chen) [2]. These compounds are found in various plant parts, including roots, stems, leaves, and fruits. From a chemical structure perspective, these bitter components belong to extremely different categories, mainly including alkaloids (such as berberine), terpenoids (such as acanthine), flavonoids (such as naringin), cycloenether terpene glycosides, and steroidal saponins, etc., demonstrating a high degree of diversity in their chemical skeletons. Despite their significant structural differences, they are all perceived through interaction with TAS2Rs. Studies have shown that when different chemical classes of bitter compounds activate TAS2Rs, they often rely on certain common molecular features, such as hydrophobic groups, hydrogen bond donors/acceptors, and planar aromatic systems, etc. These features enable them to be recognized by the same or different TAS2R subtypes in multiple binding modes. This thus forms a complex structure-taste relationship [19]. There exists a significant association between the traditional therapeutic effects of medicinal food plants and their bitter compounds, which will be discussed in the following sections.

### 2.2. Traditional Uses of Bitter Medicinal Food Plants

The “Su Wen: On the Correspondence of Yin and Yang” states that “sour and bitter flavors correspond to Yin,” indicating that the bitter taste belongs to Yin and thus has the properties of draining, descending, and solidifying: draining can be categorized into descending drainage, clearing drainage, and unblocking drainage; descending serves to guide stagnation through the bowels; and solidifying has the functions of draining fire while preserving Yin and reinforcing the kidney’s Yin. The same text also states that “fire produces bitterness,” which means that the bitter taste is associated with fire, and therefore possesses the properties of drying, warming, and dispersing: drying can treat dampness, warming can disperse cold, and dispersing can release fire. The bitter components found in medicinal food plants serve as the material carriers of this system of properties and effects. From a modern biomedical perspective, these traditional functional descriptions broadly correspond to the regulation of glycolipid metabolism, inflammatory response, gastrointestinal motility, and immune homeostasis. Bitter taste receptors (TAS2Rs), widely distributed in extraoral tissues, serve as the key molecular link between bitter compounds and these physiological processes. The bitter components in medicinal food plants serve as the material carriers of the efficacy system based on taste. The following section classifies the main bitter medicinal and edible plants according to their traditional therapeutic uses, and discusses them in conjunction with modern biochemical evidence.

#### 2.2.1. Heat-Clearing and Detoxifying Herbs

The basis of the bitter taste’s heat-clearing effect lies in its yin and cold nature. Classics indicate that although bitterness arises from fire, it ultimately belongs to yin, which endows bitter cold medicines with the unique ability to directly subdue fire toxins and clear heat while preserving yin. The power of bitterness to dry wet stems from its nature of fire. The “Inner Canon of Huangdi: Discussion on the Timing of Organs and Qi” states, “The spleen is damp; quickly consume bitterness to dry it,” and the “Inner Canon of Huangdi: The True Essentials” also mentions that “dampness is excessive within” and “excessive dampness should be treated with bitterness.” Yixue Zhongzhongshen Xilu (Medical Connoisseurship) states: “Bitterness is the flavor associated with Fire, and dryness is the quality attributed to Fire,” thereby elucidating the intrinsic relationship between bitterness and dryness through the theoretical framework of the homology of flavor and nature in traditional Chinese medicine.

The therapeutic strategy of clearing heat and draining dampness, combined with drying dampness, establishes the foundational approach for clearing heat, drying dampness, reducing fire, and detoxifying. *Coptis chinensis* Franch., a typical representative first recorded in the “Shennong Bencao Jing”, is characterized by its bitter taste and cold nature. It is a crucial medicine for treating damp-heat diarrhea and dysentery. Traditionally, it has been used for conditions such as damp-heat fullness, vomiting, diarrhea, jaundice, excessive heart fire, and sores, abscesses, and toxic swellings. Representative formulas include Xiang Lian Wan and Huang Lian Jie Du Decoction. The berberine-type alkaloids contained, such as berberine, coptisine, and palmatine, serve not only as a source of intense bitterness but also as the material basis for antibacterial and anti-inflammatory effects [20,21]. Recent studies have confirmed that berberine exerts dual effects by activating the TAS2R38 receptor: it promotes the secretion of GLP-1 from intestinal L cells through the PLCβ2-PKC pathway, thereby regulating glucose and lipid metabolism in response to the “dampness-drying” effect; simultaneously, it inhibits the MAPK/NF-κB pathway to regulate macrophage polarization, alleviating inflammatory responses to support the “heat-clearing and fire-dispelling” action [22,23].

*Andrographis paniculata*, known for its similar effects, has a bitter taste and a cold nature. It is particularly effective in clearing heat, detoxifying, cooling the blood, and reducing swelling. It is widely used for treating wind-heat colds, sore throats, and lung heat coughs. The diterpene lactones, such as andrographolide, contained in Andrographis paniculata contribute to its bitterness and primary medicinal efficacy [24]. The same bitter herb, *Sophora flavescens*, possesses the functions of clearing heat, drying dampness, exterminating insects, and promoting diuresis. In the “Synopsis of Prescriptions of the Golden Chamber,” the *Sophora flavescens* decoction is used to treat fox-possessed diseases. The quinolizidine alkaloid, matrine, contained in it is a representative bitter component [25]. Research indicates that matrine can upregulate intracellular Ca^2+^ concentration through the Gαgust-PLCβ2-IP3 pathway, stimulating GLP-1 secretion. This provides a molecular basis for its metabolic regulatory effect of clearing heat and drying dampness.

In addition to anti-inflammatory and metabolic regulation, bitter compounds such as berberine, andrographolide, and oxymatrine can modulate tumor-related pathways like NF-κB and PI3K/Akt by activating different subtypes of TAS2Rs, thereby inhibiting tumor cell proliferation and migration. This aligns with the traditional understanding of the anti-tumor properties of bitter flavors, which are believed to ‘clear heat and detoxify, and dispel toxins and masses.’ However, current research predominantly focuses on the in vitro interactions between single active ingredients like berberine and oxymatrine and specific TAS2R subtypes, making it difficult to fully capture the overall effects of traditional compound formulas with multiple components.

#### 2.2.2. Wind-Dispersing and Stagnation-Removing Herbs

Bitterness is associated with the Yin nature and possesses a downward movement, thus facilitating the expulsion of stagnation. The “Medical Mirror of Gu Songyuan” states, “If dryness causes blockages, then evil is solidified within; therefore, it should be treated with bitterness to promote downward movement.” This highlights the essential principle of bitterness in facilitating the expulsion of stagnation. The idea of “bitterness promotes downward movement” refers to the ability of bitter flavors to drive intestinal blockages out from below. Furthermore, bitterness also has the effect of “dissolving blockages,” as seen in the action of seaweed in breaking down and expelling accumulations, which is an extension of the principle of promoting expulsion. Within the realm of medicinal food plants, some bitter plants, although not as aggressive as strong purgatives like rhubarb or Glauber’s salt, similarly leverage their bitter nature to promote intestinal lubrication and relieve constipation, or to regulate qi and alleviate stagnation.

Kuding tea, characterized by its bitter and sweet flavor, has a cooling effect, disperses wind-heat, clears the head and eyes, and alleviates thirst. Its bitterness is derived from two main classes of compounds: triterpenoid saponins and polyphenols [9,26]. The raw and processed products of *Polygonum multiflorum* exhibit significant differences in bitterness and efficacy: the raw product is notably bitter and can moisten the intestines and relieve constipation, commonly used for intestinal dryness and constipation; after steaming, the bitterness is greatly reduced, and it specializes in nourishing the liver and kidneys, as well as benefiting essence and blood. Studies have shown that the laxative effect of the raw product is closely related to polyphenolic components such as epicatechin and proanthocyanidins B1/B2, which decrease in content after processing, leading to a corresponding reduction in bitterness [27]. Commonly used for breaking qi and resolving accumulation, as well as transforming phlegm and relieving distension, both Zhi Qiao (the dried immature fruit of *Citrus aurantium*) and Zhi Shi (the mature fruit) have a bitter, pungent, and sour taste. They are commonly employed in the treatment of chest and abdominal distension and constipation due to food accumulation. The flavonoid glycosides contained in them, such as naringin and hesperidin, serve as both the bitter-tasting components and the pharmacological basis for their qi-regulating and stagnation-relieving effects [28]. Among them, active products such as naringin can act as TAS2R14 agonists, regulating gastrointestinal motility and digestive secretion through intestinal bitter taste receptor signals, providing a receptor-level explanation for its traditional function of regulating qi and promoting digestion.

#### 2.2.3. Interior-Warming and Lung-Descending Herbs

Bitterness is associated with the Yin element, characterized by its descending nature, which facilitates the downward release of lung qi. As stated in the “Su Wen: On the Timing of Organ Qi,” “when lung bitterness causes qi to rise, one should urgently consume bitterness to release it.” The proper functioning of lung qi relies on its ability to descend; thus, the downward movement of bitterness helps to counteract the upward rebellious qi, leading to relief from coughing and wheezing. On the other hand, bitterness is associated with fire; mild bitterness can warm and promote. The “Medical Records of Traditional Chinese Medicine and Western Medicine” clearly states, “Bitterness is the flavor of fire, while dryness is the nature of fire.” Li Shizhen noted that the bitterness of Bupleurum helps to reduce fever, as it is “bitter and promotes; it is a marker for dispersing fire.” Although Ephedra is bitter, it can induce sweating and elevate the qi, as it is regarded as “the thin flavor, the Yang within Yin.” Such medicinal substances excel in warming and dispersing, showcasing a different functional dimension of bitterness that contrasts with the cooling and descending qualities associated with fire.

*Zanthoxylum schinifolium* is characterized by its pungent, warm, and slightly bitter taste, which helps to dispel cold from the middle, relieve pain, and eliminate parasites. It is used for cold pain in the abdomen, vomiting, diarrhea, and abdominal pain due to parasitic accumulation. The bitterness primarily comes from 7-methoxycoumarin, a component that can activate hTAS2R14 to mediate the sensation of bitterness [29]. *Artemisia argyi* is characterized by its bitter and warm properties, which help disperse cold, alleviate pain, warm the meridians, and stop bleeding. It is a crucial herb in gynecology for regulating menstruation and moxibustion therapy. The exceptionally strong bitterness of mugwort is attributed to the presence of sesquiterpenes such as 1α,6α,8α-trihydroxy guaiolide and artemisinin derivatives [30]. Bitter almonds are slightly warm and have a bitter taste, containing a small amount of toxicity. They are essential for descending Qi, stopping cough, and relieving asthma. The amygdalin contained in bitter almonds, as a cyanogenic glycoside, contributes both to the bitterness and to the medicinal efficacy [31].

Modern research has provided molecular evidence for the “lung-lowering and asthma-relieving” effect of bitterness: various bitter active ingredients can exert anti-inflammatory and asthma-relieving effects through TAS2Rs expressed in the airways. Saikosaponin B, as a specific agonist of TAS2R14, can inhibit IGE-induced mast cell degranulation. Baicalin and quercetin A inhibit the release of inflammatory factors induced by lipopolysaccharide through the TAS2R4/TAS2R14 pathway, ultimately alleviating airway inflammation and reducing airway hyperresponsiveness [32,33].

#### 2.2.4. Summer-Heat Clearing, Detoxifying, and Tonic Herbs

Bitterness is associated with Yin and can strengthen Yin fluids and Kidney Yin. The “Suwen: Discussion on the Seasonal Laws of the Zang and Qi” first advocates that “bitterness can strengthen Yin.” The “Suwen: Discussion on the Seasonal Laws of the Zang and Qi” further states, “If the Kidney desires to be strengthened, one should urgently consume bitterness to strengthen it, using bitterness to supplement it.” The “Compendium of Materia Medica” interprets this as “bitterness can drain heat and strengthen the Kidney, with draining containing supplementation.” The large Yin-replenishing pill made from *Anemarrhenae Rhizoma* and *Phellodendri Cortex* is a practical example. This principle of “using draining as supplementation” is equally applicable to food and medicinal plants.

*Momordica charantia* has a bitter taste and a cold nature, and is known for its ability to clear heat, dispel summer heat, improve eyesight, and detoxify. In traditional medicine across various regions, it is commonly used to treat conditions such as diabetes and carbuncles. Its characteristic bitter taste is attributed to cucurbitane-type triterpenoids, such as momordicin I and its propanoyl derivatives [34,35]. Among them, cucurbitacin B can regulate the Gαgust-PLCβ2-IP3 pathway by activating TAS2R10, induce the release of GLP-1 and insulin, and improve the hyperglycemic state, providing mechanism support for its traditional hypoglycemic application [36]. Both Ginseng and Notoginseng are representative tonic herbs characterized by their sweet yet slightly bitter taste. Ginseng is known for its ability to replenish vital energy, while Notoginseng is recognized for its effectiveness in promoting blood circulation and alleviating pain. The slightly bitter flavor is closely associated with the presence of compounds such as Ginsenosides Rb1 and Rg1 [37,38].

In summary, the medicinal food plants characterized by their bitter taste address a variety of syndromes, including heat-clearing, purgation, warming the middle, and tonifying. The bitter compounds found within these plants can be classified into four major chemical categories: alkaloids, phenolics, terpenes, and glycosides. This correspondence reveals the chemical basis of the traditional efficacy of bitter-tasting medicines from the perspective of ethnopharmacology.

### 2.3. Classification of Bitter Compounds

#### 2.3.1. Alkaloids

Alkaloids are nitrogen-containing organic compounds primarily derived from plants [39]. Their molecules typically contain one or more nitrogen atoms, most of which are located within heterocyclic structures. These compounds usually have a distinct bitter taste and often exhibit significant biological activity or potential toxicity [40]. Moderate intake of certain alkaloids may provide health benefits, whereas excessive intake can be harmful. This contrast reflects the evolutionary significance of bitterness as a warning signal for potentially harmful substances.

In traditional Chinese medicine, alkaloids constitute an important group of bitter-tasting active ingredients [41]. For example, the berberine-type alkaloids found in *Coptis chinensis* (such as berberine, coptisine, and palmatine) represent the most characteristic group of bitter alkaloids [20]. Studies using electronic-tongue technology combined with chemical analysis have shown that the “aftertaste-bitterness” of *Coptis chinensis* is positively correlated with its berberrubine content. In contrast, “astringency” and “aftertaste-astringency” are significantly associated with alkaloids such as coptisine, epiberberine, berberine, and palmatine [21]. Furthermore, berberine and coptisine have been identified as novel agonists of the broad-spectrum TAS2R46. This finding explains their strong bitter characteristics at the molecular level [42]. Another representative alkaloid, matrine, is extracted from the leguminous plant *Sophora flavescens* and belongs to the quinolizidine alkaloids. Its bitter taste is not mediated through the classical TAS2Rs but through specific activation of the calcium-sensing receptor (CaSR), which promotes GLP-1 secretion. This finding highlights the diversity of signaling pathways involved in bitter-taste perception [25]. *Lycium barbarum* L. is a substance used both as medicine and food. Its bitterness primarily originates from docosyl-polyamine derivatives. Although these compounds are not typical alkaloids, they still illustrate the structural diversity of bitter compounds and their close association with health benefits, including antioxidative and antidiabetic effects [43].

Alkaloids also act as important bitter constituents in MFPs. For example, caffeine, theobromine and theophylline, a class of purine alkaloids abundant in tea and cocoa materials, are well-known bitter compounds traditionally used both as dietary substances and herbal resources [28]. Quinine, a quinoline alkaloid derived from cinchona trees, is often used as a standard reference compound for evaluating bitterness because of its strong bitter taste [44].

#### 2.3.2. Phenolic Compounds

Phenolic compounds are an important class of plant secondary metabolites. Its structural feature lies in the fact that one or more hydroxyl groups are attached to the aromatic ring, among which the number, position and glycosylation modification of hydroxyl substitutions are the core structural factors regulating its bitter taste intensity and the activation ability of the TAS2R receptor. Phenolic hydroxyl groups can bind to bitter taste receptors such as TAS2R14 and TAS2R39 through hydrogen bonding. Generally, the higher the degree of mother nucleus hydroxylation, the stronger the receptor activation ability and bitter taste intensity. The type of glycogroups and their connection sites can significantly affect bitter activity by altering molecular polarity and receptor affinity [45]. These compounds are widely distributed in medicinal food plants, and serve as key constituents responsible for their organoleptic properties, particularly bitterness. The bitterness of phenolic compounds usually arises from their interaction with salivary glycoproteins, which alters oral tactile and taste perception. Common plant phenols include flavonoids, phenolic acids, lignans, and tannins [46].

The characteristic bitterness of citrus fruits, such as pomelo and lime, primarily originates from flavanone glycosides, particularly naringin and neohesperidin [28]. Both are direct bitter-causing components that have been clearly confirmed through monomer sensory evaluation and receptor experiments. Their bitter activity is directly related to the neohesperidose group linked at the C-7 position. When the same aglycone is linked to rutin, there is no obvious bitter taste. These compounds occur in relatively high amounts in both the peel and flesh of the fruit. They are also the primary bitter constituents of the traditional Chinese medicinal materials *Fructus aurantii* and *Fructus aurantii immaturus*.

The bitterness of *Polygonum multiflorum* Thunb. is closely linked to its polyphenolic profile. Its main bitter constituents include epigallocatechin gallate, catechins, proanthocyanidin B1, proanthocyanidin B2, and epigallocatechin. Research has shown that reducing the levels of these polyphenols during processing significantly decreases bitterness intensity [27]. The contribution to bitterness was verified at the component level, but the independent bitter-inducing activity of each monomer still needs further verification. This finding indicates that the bitter attribute of the material has a clear chemical basis.

Phenolic compounds are also closely related to bitterness in many plant-derived medicinal beverages, such as red wine. Studies have identified caffeic tartaric acid, kaempferol, and quercetin-3-O-rutinoside as potential contributors to bitterness, whereas ethyl protocatechuate is negatively associated with bitterness. Further analysis using a partial least squares regression (PLSR) model showed that anthocyanin derivatives and quercetin glycosides contribute more substantially to bitterness. In contrast, monomeric and dimeric flavanols—traditionally regarded as key contributors—showed relatively minor effects [47].

#### 2.3.3. Terpenoids

Terpenoids are a large and structurally diverse class of natural products derived from isoprene units. They are widely distributed in various medicinal plants. Terpenoids can be classified into subtypes based on the number of isoprene units, including monoterpenoids, sesquiterpenoids, diterpenoids, and triterpenoids. These compounds are important compounds of plant secondary metabolism.

In traditional Chinese medicine, *Andrographis paniculata* (Burm.f.) Nees contains diterpene lactones, such as andrographolide, neoandrographolide, 14-deoxyandrographolide, and dehydroandrographolide, which are identified as the main bitter compounds [24]. Similarly, the bitter taste of *Taraxacum mongolicum* is primarily attributed to the sesquiterpene lactone compounds it contains. These compounds contribute to its strong bitterness [48].

The strong bitter taste of *Artemisia princeps* Pamp. is derived from two unique sesquiterpene compounds: 1α,6α,8α-trihydroxy-5α, 7β-guaia-3,9,11(13)-trien-12-oic acid and its lactone derivative, artesin. Research has shown that its bitterness intensity is even greater than that of caffeine [30]. Additionally, compounds such as 7-ketologanin, sweroside, and loganin identified in *Lonicera caerulea* L. belong to the iridoid class, commonly found in the leaves and roots of various medicinal plants. These compounds exhibit typical bitter taste attributes [49].

The bitterness of bitter gourd is primarily attributed to cucurbitane-type triterpenoids, including 3-O-malonylcucurbitacin I, cucurbitacin I, and (23*E*)-3β-O-malonyl-7β,25-dihydroxycucurbitacin 5,23-diene-19-aldehyde [35]. These compounds collectively contribute to its strong bitterness.

Although the bitterness in *Humulus lupulus* is not entirely derived from terpenoids, its bitter acid compounds (such as α-acid and β-acid) provide typical bitterness in beer brewing and exhibit various biological activities, including antibacterial and anti-inflammatory effects [50]. Therefore, it holds significant importance in both industrial and pharmacological research.

#### 2.3.4. Glycosides

Glycosides are compounds formed by attaching sugars or sugar derivatives to non-sugar substances (aglycones) via terminal carbon atoms. Its bitter taste perception and biological activity are jointly regulated by the aglycone mother nucleus and the sugar chain structure: The type of aglycone skeleton (such as triterpenoids, steroids, and cyanoaglycones) is the core structural basis for binding to bitter taste receptors and generating bitterness. The quantity, type and connection sites of sugar groups regulate the receptor binding affinity by changing the molecular polarity and steric hindrance, thereby influencing the intensity of bitterness. They also directly determine the solubility characteristics and in vivo biological effects of the compound. Among them, saponins are a class of complex-structured glycoside compounds that are widely distributed in the plant kingdom. The combination of triterpenoids or steroid aglycones with polysaccharide chains generally gives them a distinct bitter taste.

The bitterness in *Panax ginseng* primarily results from ginsenoside compounds, including Rb1, Rg1, Rg2, Rf, and Rb3, with ginsenoside Rb1 contributing the most intense bitterness [37]. The bitterness intensity of this type of damane-type triterpene saponins is usually related to the number of sugar chain substitutions. Rb1, due to its longer sugar chain and higher degree of substitution, shows a more pronounced bitterness. Similarly, the primary bitter compounds of *Panax notoginseng* are ginsenosides Rg1, Rb1, and Rd [38]. The bitterness in Kuding tea is primarily attributed to its triterpene saponins [26], which are also key active compounds in this medicinal material.

Amygdalin, the main bitter component in *Semen armeniacae*, is a cyanogenic glycoside. It demonstrates pharmacological potential in antitussive, anti-inflammatory, anti-tumor, and neuroprotective activities. However, its cyanogenic glycoside structure can be hydrolyzed to release hydrogen cyanide, a mitochondrial respiratory chain inhibitor with potential toxicity, thus its dosage must be strictly controlled in clinical applications to prevent adverse reactions [31].

At present, there is no universal quantitative rule formed between the intensity of bitterness and biological activity/therapeutic efficacy. This is mainly constrained by three factors: the evaluation criteria for bitter taste intensity are not uniform (sensory scores, taste thresholds, etc., are mixed), the heterogeneity of biological activity endpoints is strong (in vitro, in vivo and clinical indicators are difficult to directly correspond), and the components of medicinal plants are complex, and the quantitative rules of monomers cannot be simply extrapolated to the overall extract. Therefore, although bitterness can indicate the presence of active ingredients, its intensity itself cannot be used as a reliable quantitative indicator of drug efficacy.

## 3. Separation and Screening Strategies for Bitter Compounds

**Figure 3:**
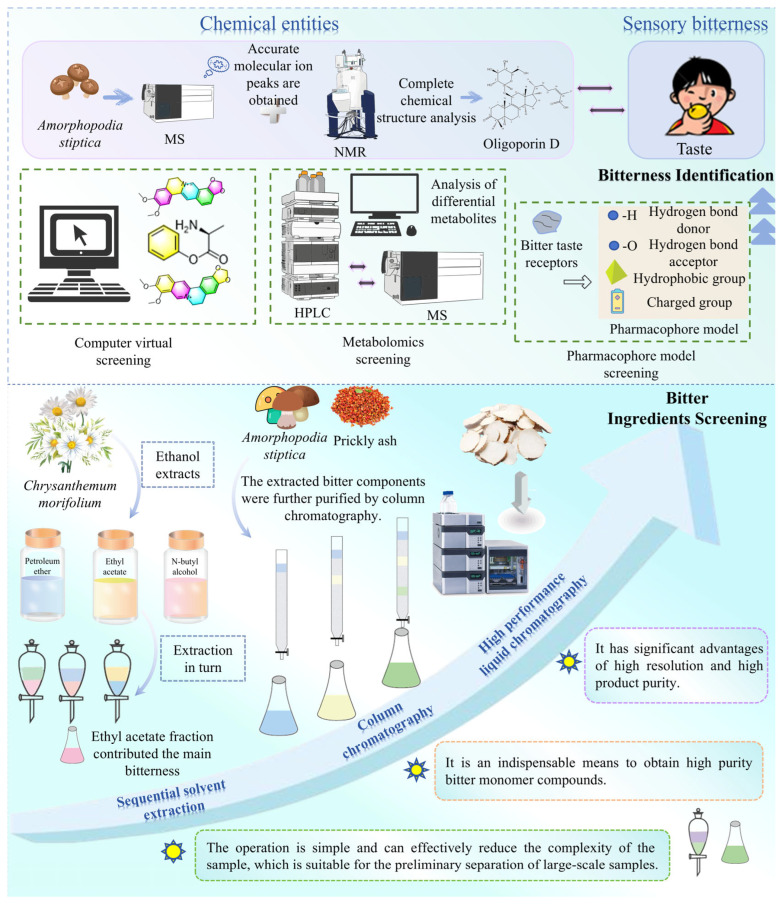
Flowchart of strategies for the separation, screening and identification of bitter compounds.

Bitterness is a common organoleptic property in natural products and herbal medicines. Excessively intense bitterness, however, markedly reduces palatability and acceptability, thereby limiting their practical application in phytotherapy and traditional medicine. Therefore, the efficient and precise isolation, identification and modulation of bitter compounds hold considerable significance for ethnopharmacological research and herbal drug development. This section systematically reviews current strategies for separating and screening bitter compounds (Figure 3, Table 1). Traditionally, activity-guided fractionation combined with sensory evaluation has been the primary approach for identifying and isolating bitter compounds. In recent years, the integration of computer-aided screening technologies with various omics methods has driven the field toward higher throughput and greater automation. The following section elaborates on the principles, operational procedures, application advantages, and current limitations of these methods.

### 3.1. Separation and Purification Techniques for Bitter Compounds

#### 3.1.1. Sequential Solvent Extraction

Sequential solvent extraction is a widely used method for separation based on differences in compound polarity. This method enriches target compounds by successively extracting samples with solvents of varying polarities and grouping compounds based on their polarity ranges. Common organic solvents used for extracting bitter compounds include petroleum ether [29], dichloromethane [35], ethyl acetate [51], n-butanol, and ethanol [52]. Gradient extraction of the crude extract, from low to high (or reverse) polarity, allows for the initial separation of compounds based on polarity, effectively enriching bitter compounds in a specific range. Following this initial separation, sensory evaluation is commonly employed to guide the separation strategy.

For example, in the study of *Chrysanthemum morifolium*, the ethanol crude extract was successively extracted with petroleum ether, ethyl acetate, and n-butanol. Sensory analysis revealed that the ethyl acetate fraction contributed the primary bitterness, making it the key fraction for the subsequent separation and identification of bitter compounds [53]. In the study of *Momordica charantia* L., researchers extracted freeze-dried samples with 75% methanol and then separated them into five compounds—n-hexane, dichloromethane, ethyl acetate, n-butanol, and water—through liquid–liquid extraction. Sensory evaluation results indicated that n-butanol and dichloromethane extracts exhibited a significant bitter taste [35], suggesting that these compounds serve as the main carriers of bitter taste characteristics.

The advantage of sequential solvent extraction is its simplicity, making it suitable for the preliminary separation of large-scale samples. This method effectively reduces sample complexity and improves the efficiency of subsequent analyses. However, this method has limited separation capabilities for bitter compounds with similar polarities and structures. Therefore, in complex systems, it is often combined with high-resolution separation techniques to achieve more precise component analysis.

#### 3.1.2. Column Chromatography Techniques

Column chromatography is a core technology for the separation and purification of natural products. Its principle involves separating compounds in a mixture based on differences in adsorption, distribution, ion exchange, or molecular size between the stationary and mobile phases. Column chromatography can be classified into various types based on the properties of the stationary and mobile phases, including silica gel, reversed-phase, gel, and ion-exchange chromatography. In bitter compound studies, researchers select chromatographic strategies based on the physicochemical properties of the target compounds to achieve efficient separation and enrichment.

After initial extraction and enrichment, the focus of bitter compound identification shifts to refined separation and purification. Column chromatography is central to this process, serving as the foundation for subsequent structural analysis and activity verification. Several commonly used column chromatography techniques for separating bitter compounds and their applications are summarized below:

(1) Silica gel column chromatography: This method separates compounds based on polarity differences and is widely used for preliminary and crude separation. For instance, in the study of bitter compounds in *Zanthoxylum bungeanum* Maxim, the petroleum ether extract with the strongest bitter activity was further subdivided using silica gel column chromatography. This provided a key pretreatment basis for subsequent thin-layer chromatography and ultra-performance liquid chromatography-quadrupole-time-of-flight mass spectrometry (UPLC-Q-TOF-MS) identification [29].

(2) Macroporous adsorption resin column chromatography: This method is suitable for the adsorption and desorption of macromolecules or strongly polar compounds, commonly used in the extraction and purification of traditional Chinese medicine. In the study of bitter compounds in peony seeds, the n-butanol extract was separated using an AB-8 macroporous resin column and eluted in a gradient with ethanol-water solutions of varying concentrations. Quantitative sensory evaluation indicated that bitter compounds were mainly enriched in the 30% and 50% ethanol elution fractions [54].

(3) Gel column chromatography: This method separates compounds based on differences in molecular size. In the study of bitter compounds in *Amaropostia stiptica* mushrooms, the active fractions initially separated by silica gel columns were further purified using Sephadex LH-20 gel columns. Sensory evaluation confirmed the bitterness of each sub-fraction, allowing for targeted tracking and enrichment of the desired compounds [55].

The aforementioned chromatographic techniques effectively separate compounds based on differences in polarity, molecular size, and hydrophobicity, making them indispensable for obtaining high-purity bitter monomer compounds. However, column chromatography is time-consuming and requires large amounts of organic solvents. It has limitations in the enrichment and separation of trace bitter compounds and often requires a combination with other high-resolution analytical methods to improve separation efficiency.

#### 3.1.3. High-Performance Liquid Chromatography Technology

High-performance liquid chromatography (HPLC) is a key technology for separating complex mixtures and obtaining high-purity compounds. It is widely preferred for its high separation efficiency, sensitivity, and reproducibility. In the HPLC system, medium-pressure liquid chromatography (MPLC) achieves separation efficiency and flux between conventional column chromatography and analytical HPLC, making it suitable for the rapid classification of complex samples. Preparative HPLC (prep-HPLC) serves as a large-scale extension of analytical HPLC. Analytical HPLC establishes separation methods and verifies product purity, while prep-HPLC enables efficient preparation of target substances on milligram to gram scales by increasing column diameter and flow rate, providing high-purity samples for subsequent structural analysis and activity studies [56].

In the systematic identification of key bitter compounds in *Angelica dahurica*, component profiles were first obtained using analytical HPLC-DAD. Subsequently, compounds corresponding to each chromatographic peak were collected on a large scale using prep-HPLC, and their bitterness intensity was immediately evaluated through sensory analysis. This process enabled rapid identification of the key bitter fractions [57]. Similarly, in the study of bitter compounds in bitter gourd, researchers combined preparative chromatography and sensory evaluation techniques: First, the active n-butanol fraction was initially classified by MPLC. A bitterness intensity map was generated using taste dilution analysis to locate the core bitter compounds. Next, prep-HPLC was employed for further separation. Throughout the process, taste dilution analysis was continuously employed to monitor the bitterness of the secondary fraction. This approach enabled precise orientation of the separation process [35].

While HPLC technology offers significant advantages, such as high resolution and product purity, its throughput is limited, and the high cost of processing large sample volumes restricts its large-scale application. Furthermore, the sensory-guided fractionation strategy based on this technology still has inherent limitations in the bitter taste tracking of complex phytochemical matrices. Firstly, human sensory evaluation itself has significant individual differences and subjectivity. The perception threshold and intensity score of the same bitter substance by different evaluators may fluctuate greatly, which directly affects the reproducibility of key steps such as taste dilution analysis. Secondly, the masking, synergistic or inhibitory effects among the matrix components in plant extracts may lead to sensory-guided fractionation directed towards non-critical components or the omission of truly active compounds [58].

For this reason, various chromatographic techniques and evaluation methods are usually not used in isolation but integrated into a comprehensive separation strategy. To a certain extent, this compensates for the shortcomings of a single method, providing a reliable foundation for subsequent structural identification and functional research, while taking into account both separation efficiency and sample purity. Future research still needs to further optimize the existing separation strategies. On the one hand, it is necessary to reduce the bias caused by complex matrices through the combination of multiple techniques. On the other hand, objective evaluation methods, such as in vitro receptor activation experiments, should be combined to make up for the deficiencies of subjectivity and variability in human sensory evaluation, and improve the accuracy and reliability of bitter substance identification.

**Table 1:** Methods for separation and identification of bitter compounds in extracts of medicinal food plants.

Types	Plant Part	Extraction Method	Purification Technique	Identification Techniques	Bitter Taste Evaluation Method	Bitter Substances/Main Findings	References
*Amomum villosum* L.	Fruit	Water, 50% Ethanol	HPLC-DAD, prep-HPLC	UPLC-Q-TOF-MS	Electronic tongue	Catechin (primary).	[15]
*Amaropostia stiptica*	Fungal fruiting body	80% Methanol	Extraction grading, Silica gel column chromatography, Semi-prep HPLC	HR-ESI-MS, NMR	Sensory evaluation panel	Five triterpene glycosides: Oligoporin A, B, D–F.	[55]
*Idesia polycarpa* var.	Fruit	75% Ethanol	prep-HPLC, Semi-prep HPLC	UPLC-Q-TOF-MS, NMR	Sensory evaluation panel	Five key compounds: Pyrocatechol monoglucoside, Demethylconiferin, Idesin, Idescarpin, Idevestioside.	[59]
*Momordica charantia* L.	Fruit	75% Methanol	Extraction grading, Macroporous adsorbent resin, MPLC, prep-HPLC	HR-ESI-MS, NMR	Sensory evaluation panel	Primary bitter compounds: 3-O-Malonylmomordicine I, Momordicine I, (23E)-3β-O-Malonyl-7β,25-dihydroxycucurbita-5,23-dien-19-al. Momordicin K is an artifact formed during extraction.	[35]
*Lycium barbarum* L.	Fruit	50% Ethanol	MPLC, Macroporous Resin Separation, Prep-HPLC,	HR-ESI-MS, NMR	Sensory evaluation panel	Seven diacylpolyamine derivatives: lyciamarspermidine A–C, lyciamarspermine A, B, lycibarbarspermidine A, L.	[43]
*Panax ginseng C. A.* Mey.	Root	Water, 55% Ethanol	HPLC-DAD, Prep-HPLC	UPLC-Q-TOF-MS	Electronic tongue	Bitterness from five ginsenosides: Rg1, Rf, Rb1, 20(S)-Rg2, Rb3. Rb1 is the primary contributor.	[37]
*Panax notoginseng (Burk) F. H. Chen*	Root	Water	Extraction grading, Macroporous adsorbent resin, HPLC	UPLC-Q-Orbitrap HRMS	Sensory evaluation panel, fNIRS	Primary contributors: ginsenosides Rg1, Rb1, Rd.	[38]
*Zanthoxylum bungeanum* Maxim.	Fruit peel	70% Ethanol	Extraction grading, Macroporous, Silica gel column chromatography, TLC	UPLC-Q-TOF-MS	Sensory evaluation panel	Key bitter compounds: 7-Methoxycoumarin (predominant) and 8-Prenylkaempferol.	[29]
*Zanthoxylum schinifolium* Sieb.	Fruit peel	70% Ethanol	Prep-HPLC	UPLC-Q-TOF-MS	Sensory evaluation panel	Quercetin, kaempferol, and isorhamnetin are critical due to high levels and low bitterness thresholds.	[53]
*Ziziphus jujuba* cv.	Dried fruit	95% Ethanol	Extraction grading, Macroporous resin	LC-MS/QTOF	Sensory evaluation panel	35 potential bitter compounds identified; rutin is the key contributor.	[60]

### 3.2. Screening and Discovery Strategies for Bitter Compounds

After completing the structural characterization of the compound, its bitter taste properties must be further evaluated to clarify its taste profile. Traditional sensory evaluation methods often face efficiency bottlenecks, making them inadequate for large-scale compound screening [61]. With advancements in modern analytical techniques, efficient and precise screening strategies for bitter compounds have steadily matured, including computer virtual screening, bioaffinity techniques, and physicochemical detection methods. These strategies can rapidly identify potential bitter substances or ligands that bind to bitter receptors from large compound libraries, significantly accelerating the discovery and identification of bitter compounds.

#### 3.2.1. Computer-Aided Virtual Screening Technology

With the rapid development of artificial intelligence and the growing availability of chemical data, computer-aided methods have become efficient, cost-effective, and reliable tools for predicting the bitter properties of compounds [14]. Current computational strategies for predicting bitter compounds include machine learning methods and virtual screening based on ligands and structures.

Machine learning, a key branch of artificial intelligence, plays an increasingly important role in chemoinformatics. These models rely on large molecular databases and use supervised or unsupervised learning to extract patterns from chemical features, aiding in molecular property prediction and compound design [62]. As the study of bitterness deepens, the number of identified bitter compounds continues to increase, prompting the development of efficient computational screening models to accelerate evaluation and create comprehensive bitter compound databases (Table 2).

BitterDB is a public database that systematically collects bitter compounds. Following its 2024 update, it now includes 2250 bitter compounds, 236 TAS2Rs, and nearly 1800 ligand-receptor interaction records across 66 species. It also offers tools for receptor structure linking and sequence alignment, providing strong support for cross-species comparisons and the structure-function study of bitter taste perception [12]. BitterPredict is a machine learning-based tool for predicting bitter taste. It uses known bitter compounds from BitterDB as a training set and the AdaBoost algorithm, combined with basic physicochemical descriptors, to perform “bitter/non-bitter” binary classification [63]. Its dataset includes numerous hypothetical non-bitter molecules. Another tool, e-Bitter, uses a fully experimental verification dataset, effectively avoiding the noise of hypothetical data. It innovatively combines the Extended Connectivity Fingerprint (ECFP) with a multi-algorithm consensus model, including deep learning, to further improve prediction performance [64].

The data basis and verification strategies of the three are different, and their limitations vary when dealing with structurally complex natural products. Although BitterDB has a large inclusion, it is biased towards known skeletons and has insufficient coverage of rare natural products, which restricts the generalization ability of the model. The negative sample dependence hypothesis of BitterPredict has not been experimentally confirmed, and it has a relatively high risk of false positionality for special structures such as polyhydroxy and glycosylation. e-Bitter enhances robustness with a full experimental dataset and consensus strategies, but the proportion of natural products in the training set is limited, and the prediction accuracy for trace components in complex matrices still lacks systematic verification.

While machine learning has yielded remarkable results in the binary classification of “bitter/non-bitter”, providing an efficient tool for preliminary screening, challenges remain in constructing regression models to predict “bitterness intensity”. Such models must not only identify the bitter taste characteristics of compounds but also analyze the complex and nonlinear dose–response relationship between compounds and human sensory intensity. Therefore, relying on precise quantitative data from standardized sensory panels or electronic tongue systems is essential for developing accurate bitter taste intensity prediction models.

At the mechanistic level, methods such as homology modeling, molecular docking, and molecular dynamics simulations help reveal the structural basis and dynamic processes of ligand-receptor interactions [65]. The three-dimensional structure of bitter taste receptors, constructed through homology modeling, serves as a template for molecular docking to predict the orientation, conformation, and relative affinity of ligands in the binding space [65]. For instance, in the study of bitter compounds in *Corydalis yanhuso*, virtual molecular docking based on the homology model of hTAS2R10 revealed that protoberberine-type alkaloids had the optimal binding posture and strength, suggesting they were the primary contributors to the bitterness of this medicinal material [66]. In the study of bitter taste in *Chrysanthemum morifolium*, all three compounds—eugenin, orientin, and kaempferol—effectively bound to the active region of TAS2R14. The binding fraction was greater than −8.0, surpassing that of the commonly used bitter taste reference quinine (−8.1) [54]. Its interaction relies mainly on hydrogen bonds and hydrophobic interactions, elucidating the molecular mechanism behind its bitter taste formation at the structural level.

Although knowledge-driven computer simulation methods show great potential in screening bitter substances and studying receptor interactions, they still face challenges: The existing training datasets are highly heterogeneous. It should be particularly emphasized that the binding fraction of molecular docking only reflects the theoretical binding affinity between the ligand and the receptor. It neither directly represents the functional activation effect of the receptor nor is it completely equivalent to the actual perception of bitterness in the human body. Some allosomes can bind to receptors but have no agonistic activity. Moreover, the calculation process does not take into account bioavailability, multi-receptor synergy, and interference from matrix components. The docking fraction alone is insufficient to confirm bitter taste activity. The existing training datasets have prominent heterogeneity. The active labels come from different experimental systems and the standards are not unified, which will weaken the robustness of the model’s prediction. At the same time, the data have structural biases, compound skeleton coverage bias, and some negative samples lack experimental verification, which can easily reduce the generalization ability of the model in complex matrices and increase the risk of false positives. Moreover, the scarcity of high-quality structural templates for bitter taste receptors further limits the prediction accuracy. Therefore, the verification of the experimental sensory and receptor functional levels remains an indispensable core link, used to confirm the biological reliability of the prediction results and provide data support for the iterative optimization of the model.

**Table 2:** Tools for predicting bitter compounds.

Tool’s Name	Main Role	Web Address	Advantages	Limitations and Future Development	References
BitterDB	Database of bitter compounds; prediction of bitter compounds and receptor associations.	http://bitterdb.agri.huji.ac.il (accessed on 15 June 2026)	First publicly accessible and searchable database.	Future: expand data on bitterness masking agents and inhibitors.	[12]
BitterX	Prediction of bitter compounds and their receptors.	http://mdl.shsmu.edu.cn/BitterX (accessed on 15 June 2026)	Validated recognition of novel bitter compounds and their receptors.	Limited by dataset size; expanding data will improve predictions.	[67]
e-Bitter	Machine learning-based batch prediction of bitter compounds.	https://www.dropbox.com/sh/3sebvza3qzmazda/AADgpCRXJtHAJzS8DK_P-q0ka?dl=0 (accessed on 15 June 2026)	Rapid prediction of bitterness with visual explanation of source.	Currently limited to small molecules; future: subcategorization.	[64]
BitterPredict	Prediction of bitter taste from chemical structure.	https://github.com/Niv-Lab/BitterPredict1 (accessed on 15 June 2026)	High accuracy; applicable to diverse chemical libraries.	Cannot distinguish bitterness intensity; future: subcategorization.	[63]
BitterMatch	Prediction of bitter molecule–receptor interactions using ML.	https://github.com/YuliSl/BitterMatch (accessed on 15 June 2026)	Excellent predictive performance.	Accuracy depends on quantity/quality of experimental data.	[68]
BitterSweetForest	Random Forest-based bitter/sweet	http://bioinformatics.charite.de/sweet (accessed on 15 June 2026)	First free KNIME workflow platform for prediction.	No receptor association; future: link to receptor mechanisms.	[69]
BitterSweet	Prediction of bitter/sweet taste from molecular structure.	https://cosylab.iiitd.edu.in/bittersweet/ (accessed on 15 June 2026)	Uses experimentally validated data.	Cannot predict taste of mixtures; future: more refined data.	[70]
BitterEN	Integrated model for identifying bitter peptides.	https://github.com/Shazzad-Shaon3404/Bitter (accessed on 15 June 2026)	Captures comprehensive sequence information.	Computational efficiency and predictive information potential can be enhanced.	[71]
BitterIntense	Prediction of “strong bitter taste”; analysis of toxicity-bitterness link.	Not open-source	Rapid identification of intense bitterness in early drug development.	Relies solely on chemical structure; future: integrate receptor structural data.	[72]

#### 3.2.2. Metabolomics-Based Screening Technologies

Metabolomics is a systematic analytical technique that has been widely applied in the fields of natural product chemistry and traditional Chinese medicine research. In phytochemical and ethnopharmacological investigations, it has been utilized to characterize the chemical profiles of medicinal plants and evaluate their biological effects on human health [73]. When integrated with multivariate statistical analysis, metabolomics enables reliable identification of bioactive constituents, elucidation of molecular mechanisms, and discovery of quality markers for herbal medicines [74]. The inherent properties of medicinal herbs (e.g., chemical composition, pH) and external environmental factors (e.g., temperature, oxygen exposure) frequently induce a cascade of chemical transformations, which can directly affect their organoleptic features and therapeutic stability. Accordingly, the application of metabolomics to systematically profile such changes, identify key chemical markers, and establish correlations with pharmacological properties is of substantial research significance. Notably, in the dissection of bitter and astringent taste profiles, metabolomics offers high sensitivity and throughput, allowing comprehensive capture of trace compounds that are often undetectable by conventional methods. This capability greatly advances mechanistic understanding of taste formation in medicinal herbs [15].

Current metabolomics research primarily relies on two analytical platforms: nuclear magnetic resonance (NMR) and mass spectrometry (MS). NMR offers clear advantages in targeted quantitative analysis. MS-based metabolomics is more suitable for the systematic screening and identification of unknown metabolites because of its high sensitivity and resolution [75]. Mass spectrometry is commonly combined with separation techniques. Common configurations include GC–MS, LC–MS, and CE–MS. Using ultra-high performance liquid chromatography-electrospray ionization-mass spectrometry/mass spectrometry (UPLC-ESI-MS/MS)–based metabolomics combined with the BitterDB database, 64 potential bitter compounds were identified in blue honeysuckle, covering structural types such as flavonoids, phenolic acids and terpenoids [76]. Further analysis of differential metabolites and their association with bitterness intensity identified arbutin, coumarin, and malonic acid as key metabolites regulating fruit bitterness. Another study systematically screened 37 potential bitter metabolites from *Zanthoxylum bungeanum* Maxim. through non-targeted UPLC-MS metabolomics combined with sensory-directed isolation, and identified 15 key compounds. Among these compounds, isorhamnetin showed the highest variable importance projection (VIP = 5.7) and an exceptionally low bitterness threshold (0.028 mmol/L), and was identified as a core contributor to bitterness [53].

Although metabolomics shows clear advantages in the systematic screening of bitter compounds, several challenges remain. In complex biological samples, compounds exhibit wide variation in polarity and volatility. In non-targeted analyses, a large proportion of detected metabolites remains unknown. Compared with targeted quantitative methods using reference standards, non-targeted approaches are more prone to false-positive identifications. Therefore, candidate bitter compounds identified by metabolomics must be validated by sensory evaluation, which remains the most direct and reliable method for confirming bitterness.

#### 3.2.3. Screening Techniques Based on Pharmacophore Models

A pharmacophore refers to the molecular features crucial for biological activity and their specific three-dimensional arrangement. Common features include hydrogen bond donors/acceptors, hydrophobic regions, aromatic rings, and charged groups [77]. The core of virtual screening based on pharmacophores involves converting these abstract features into three-dimensional templates, enabling rapid scanning and matching of large compound databases. Typical screening processes include constructing pharmacophore models based on known active ligands or receptor structures, generating compound databases with multiple conformations, performing three-dimensional database searches to identify matches, and prioritizing candidates for experimental verification [78].

Due to its efficiency in identifying active molecules, the pharmacophore model has become a key tool in drug discovery. This method, based on the same principle, has recently been applied in flavor science, particularly in constructing pharmacophore models targeting human TAS2Rs for the rapid identification and screening of bitter compounds in complex systems. Currently, 25 TAS2Rs are known in humans, each with different structures and ligand recognition spectra. A single receptor cannot recognize all bitter molecules, and the ligand-receptor relationship is not one-to-one [19]. For example, caffeine can be recognized by multiple receptors, including TAS2R7, TAS2R10, TAS2R43, and TAS2R46, simultaneously [79]. TAS2R39 specifically recognizes epigallocatechins and responds to theaflavins and their derivatives simultaneously [80]. To enhance screening success and efficiency, receptors with broad ligand spectra, such as TAS2R10, TAS2R14, and TAS2R46, are typically selected as models in studies [19,81,82]).

In practical applications, studies have constructed a pharmacophore model for the TAS2R14 agonist and conducted virtual screening of the Radix Bupleuri chemical component library, identifying several bupleurum saponins with high fitting values (saponins A, B, C, and D, all above 0.9), suggesting their high potential for structural matching with receptors. Subsequent cell experiments confirmed that saikosaponin B is a direct agonist of TAS2R14 [32]. Another study constructed pharmacophore models for TAS2R10, TAS2R14, and TAS2R46, successfully screening representative bitter substances, such as baicalein and berberine, from Huanglian Jiedu Decoction [83].

In research on the material basis of the bitter taste of *Panax notoginseng* [38], this method was further expanded: Using the Bitter X database and results from ultra-performance liquid chromatography-quadrupole electrostatic field orbitrap high-resolution mass spectrometry (UPLC-Q-Orbitrap HRMS), researchers selected six receptors, including TAS2R14, TAS2R40, and TAS2R10, to construct pharmacophore models for virtual screening. Based on the screening results, systematic separation and bitterness tracing, combined with sensory evaluation, ultimately identified the main bitter compounds. Through subjective and objective methods, including component knock-in experiments and functional near-infrared brain imaging, it was confirmed that ginsenosides Rg1, Rb1, and Rd are the key contributors to the bitterness of *Panax notoginseng*. This research innovatively integrates pharmacophore-based virtual screening with natural product separation chemistry, sensory science, and neuroimaging techniques, offering a novel paradigm for the precise identification of flavor compounds in complex systems.

### 3.3. Modern Analytical Methods for Identifying Bitter Compounds

After obtaining the bitter-active fraction through separation and screening, the focus of the research shifted to identifying the key flavor molecules’ structures. This stage bridges “sensory bitterness” with “chemical entities” and provides a chemical foundation for subsequent quantitative analysis, structure-activity relationship studies, and the exploration of bitterness signal transduction mechanisms. Traditional structural identification primarily relies on two techniques: NMR and MS. NMR reveals the carbon-hydrogen framework and three-dimensional configuration by analyzing the chemical environment and nuclear coupling interactions. Mass spectrometry provides precise molecular weight and fragment data based on the mass-to-charge ratio, aiding in the inference of molecular formulas and structural fragments [84]. Combining both techniques has become a standard strategy for analyzing the structure of unknown metabolites.

In studying the bitter compounds of *Amorphopodia stiptica*, researchers employed high-resolution mass spectrometry (HRMS) and multi-dimensional NMR technology [55]. First, the molecular ion peak is obtained through HRMS to infer the molecular formula. NMR analysis is then performed. The ^1^H and ^13^C NMR spectra reveal the types and numbers of hydrocarbon atoms, while COSY and HMBC spectra establish the planar structure of aglycones and confirm the glycosyl connection sites. Ultimately, spatial correlation signals in the ROESY spectra determine the relative configurations of multiple chiral centers. Thus, the chemical structure of the novel bitter triterpene saponin, oligoporin D-F, was fully analyzed. Similarly, in the study of bitter compounds in bitter gourd, the combined use of high-resolution mass spectrometry and multi-dimensional NMR (^1^H, ^13^C, COSY, HSQC, HMBC) successfully identified a series of cucurbitane-type triterpenoids [35].

High-performance liquid chromatography-diode array detection-electrospray tandem mass spectrometry (HPLC-DAD-ESI-MS/MS) provides chromatographic retention behavior, UV absorption, and mass spectrometry fragment data, making it suitable for rapid identification and quantification of compounds in complex systems. In the study of bitter compounds in *Angelica dahurica*, researchers used this technology, combined with sensory evaluation, to compare samples processed by different methods (boiling, frying, and frying followed by boiling) and identified six key bitter compounds: propoxylon oxide hydrate, bergamot lactone, piperanol, exophenin, isoexophenin, and propoxylon oxide [85].

For high-throughput identification of bitter compounds in complex matrices, UPLC-Q-TOF-MS offers significant advantages due to its high-resolution separation and precise mass determination. For example, in identifying bitter compounds in *Panax ginseng*, the study used UPLC-Q-TOF-MS to analyze bitter fractions screened by the electronic tongue. Based on quasi-molecular ions and characteristic glycogroup loss fragments in high-resolution mass spectrometry, the structure of ginsenosides was inferred and further verified using reference standards [37].

### 3.4. Sensory Evaluation Methods for Bitterness

Bitterness-induced discomfort significantly affects the acceptance and clinical application of medicinal food plants in traditional herbal practice. Whether for modulating bitterness to enhance the applicability of herbal materials in ethnopharmacological practice or for isolating bioactive compounds and verifying their functional mechanisms in basic research, systematic sensory evaluation serves as an indispensable core step. Sensory evaluation plays a pivotal bridging role in this process: it not only acts as the primary guide for the isolation and screening of bitter compounds but also serves as the ultimate criterion for assessing the efficacy of bitterness-mitigating strategies and related technical applications. Currently, sensory evaluation methods for bitterness are categorized into two types: subjective methods based on human senses and objective methods based on analytical instruments.

#### 3.4.1. Human Sensory Evaluation Methods

Relying on the human taste system to directly perceive bitterness represents a traditional evaluation method. Despite its inherent subjectivity, this method remains indispensable in the screening and verification of bitter compounds, as its results closely align with human taste perception.

The reliability of the evaluation depends on professionally trained sensory evaluators, who can accurately identify and quantify specific sensory properties through systematic training, ensuring consistency and accuracy [86]. During implementation, the recruitment and training of volunteers must adhere to relevant standards (e.g., “T/CSTM 01249-2024”), with quinine sulfate solution commonly used to establish a bitter taste scale for training. After standardizing the sample preparation, evaluators scored the anonymous encoded samples in a controlled environment, using reference samples for comparison. The data were collected repeatedly and statistically processed through multiple rounds (e.g., outlier elimination and mean calculation) to ensure result reproducibility [38].

Common methods for evaluating the contribution of bitter compounds in MFPs include: ① Dose-threshold factor (DOT), used to estimate the potential impact of known compounds [87]; ② Taste dilution analysis (TDA), a sensory tracking method to guide the discovery of potent bitter compounds during separation [88]; ③ Spectral descriptive analysis (SDAM), which is used for standardized description and intensity calibration and can verify taste interactions of key compounds in recombinant models [89]. These methods are complementary and are frequently combined in practical research. For example, in analyzing the bitter compounds of *Chrysanthemum morifolium* and *Angelica dahurica*, a comprehensive strategy combining TDA sensory tracking, electronic tongue verification, and DOT quantification was employed to efficiently locate key bitter substances [54,85].

In conclusion, the major advantage of evaluation methods based on human senses lies in their high biological relevance, providing results that closely align with real taste perception. The primary limitations include strong subjectivity, susceptibility to individual states, time consumption, and challenges in standardization and automation. Therefore, implementation requires strict control of experimental conditions and systematic training for evaluators, including professional screening.

#### 3.4.2. Instrument-Based Evaluation Methods

Electronic tongue technology simulates human taste perception by capturing taste signals through cross-sensitive sensor arrays combined with pattern recognition algorithms, and has become a representative tool for the objective evaluation of bitterness [90]. The core of this technology lies in establishing a calibration model between sensor responses and sensory evaluation data, enabling rapid and objective prediction of bitterness intensity.

At the application level, the electronic tongue can effectively distinguish and quantify bitterness among different samples using multivariate statistical methods such as PCA and OPLS-DA [91]. It is also widely used to evaluate the effectiveness of bitterness removal or taste-masking processes. Studies have shown that the electronic tongue can distinguish bitterness differences among Andrographis Herba samples from different sources [24], and verify the significant taste-masking effects of materials such as chitosan on berberine [92] and *Panax ginseng* extracts [37]. In addition, the electronic tongue is often combined with the electronic nose and GC–MS to enable systematic analysis of overall sample flavor. Another important function is its ability to monitor the taste characteristics of fractions in real time during the separation and purification of bitter compounds, providing guidance for rapid identification of target compounds. For example, this approach successfully guided the identification of catechins in *Amomum villosum* L. [93].

Although the electronic tongue offers advantages such as objectivity, high efficiency, and good reproducibility, and effectively avoids human subjectivity and fatigue, it still faces practical challenges including complex matrix interference, sensor stability, and cross-system data comparability. Future research will focus on developing novel recognition elements, promoting standardization of detection protocols and data, and integrating cloud platforms with algorithm optimization to construct intelligent sensing systems with enhanced anti-interference capability and expanded functionality.

## 4. Perception of Bitter Compounds

The previous section systematically reviewed the natural sources of bitter compounds and modern strategies for their separation and screening. These methodological advances have significantly deepened our understanding of the chemical basis of bitterness in medicinal food plants. However, to achieve a fundamental understanding and precise regulation of bitterness, it is essential to explore the biological basis of bitterness perception within living organisms. This section will focus on the transmission pathways of bitter taste signals, explaining how bitter molecules are recognized by taste receptors, transduced within cells, transmitted through nerves, and ultimately decoded into the sensation of ‘bitterness’ in the central nervous system. Clarifying this pathway not only elucidates the mechanisms underlying existing taste-masking technologies but also provides a foundation for developing rational bitterness intervention strategies based on receptor targets.

### 4.1. Bitter Taste Receptors

Bitter taste perception is primarily mediated by TAS2Rs. TAS2Rs belong to the G protein-coupled receptor (GPCR) superfamily and are widely distributed in the oral cavity as well as various extraoral tissues [94,95]. TAS2Rs have a typical GPCR topology, comprising seven transmembrane helical domains, three extracellular loops, three intracellular loops, as well as shorter extracellular N-termini and intracellular C-termini [95]. In the oral cavity, type II taste cells within taste buds serve as the primary functional units responsible for detecting the three basic tastes: sweetness, umami, and bitterness. TAS2Rs are specifically expressed on the apical microvilli of these cells, acting as a direct detection interface for bitter compounds. When bitter molecules bind to TAS2Rs, they initiate a downstream intracellular signaling cascade, ultimately activating afferent taste nerves and transmitting bitter information to the brain [96,97].

The initiation of bitter taste signal transduction relies heavily on the specific recognition between TAS2Rs and their ligands. This receptor family exhibits considerable diversity in ligand recognition, sometimes leading to confusion. There are notable differences in ligand selection among various TAS2R members. Some receptors (such as TAS2R10, TAS2R14, and TAS2R46) exhibit “broad-spectrum” characteristics [19,81,82] and can be activated by a variety of structurally distinct compounds, whereas others (such as TAS2R4 and TAS2R5) exhibit “narrow-spectrum” characteristics [19], being sensitive only to a few structurally specific ligands. This broad recognition spectrum significantly enhances the body’s ability to perceive complex chemical substances, such as plant secondary metabolites. It not only has physiological significance in detecting toxic substances but also provides the molecular basis for the initial interaction of certain bitter traditional Chinese medicine compounds, facilitating their therapeutic effects.

Recent studies have shown that TAS2Rs are not only present in the oral cavity but also widely expressed in various extraoral tissues, where they perform important physiological regulatory functions, thus significantly enhancing the understanding of their biological significance. In intestinal endocrine cells, TAS2Rs function as chemical sensors for intracellular contents, such as bile acids, bacterial metabolites, and plant alkaloids [98]. For instance, cucurbitacin B enhances AMPK activity through the Gα-gustducin and Gβγ signaling pathways by activating intestinal TAS2Rs, which promotes GLP-1 and insulin release, thus improving the hyperglycemic condition in diabetic mice [36].

TAS2Rs are expressed in respiratory ciliary epithelial cells, where they respond to harmful stimuli. Activation of TAS2Rs can trigger calcium-dependent nitric oxide (NO) production, enhance mucociliary clearance, exert direct antibacterial effects, and simultaneously promote the phagocytic activity of macrophages [94]. In immune cells such as monocytes, neutrophils, and macrophages, TAS2Rs recognize bacterial quorum-sensing molecules, regulate innate immune responses, and exert anti-inflammatory effects [94]. TAS2Rs are also expressed in the heart, thyroid, bladder, myometrium, and various cancer cells, where they regulate processes such as myocardial contraction, thyroid hormone synthesis, uterine relaxation, and tumor growth [94].

### 4.2. Activation Mechanism of Bitter Taste Receptors

**Figure 4:**
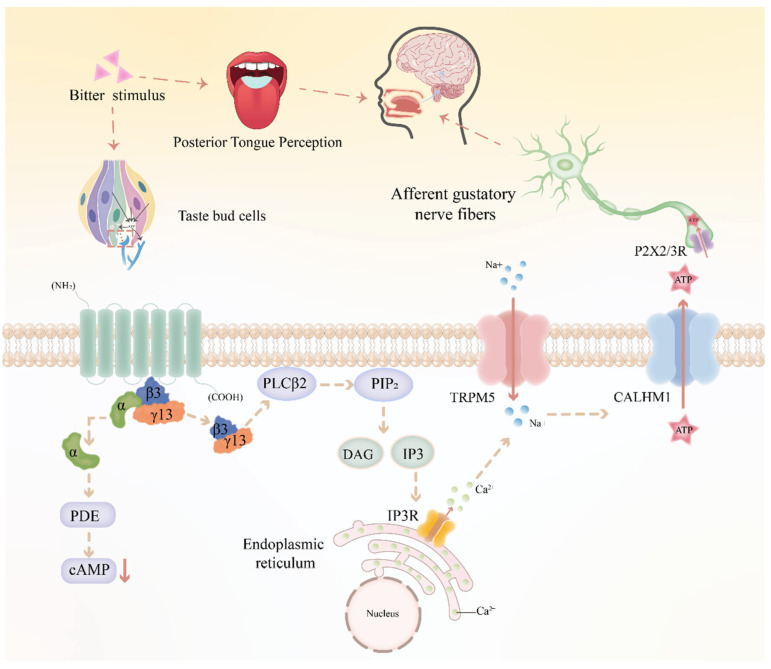
The bitter taste signal transduction pathway from taste cells on the tongue to the central nervous system in the brain.

Although TAS2Rs are expressed in various tissues and perform diverse physiological functions, this section will focus specifically on their activation process in oral type II taste cells, as the core objective of this article is to systematically explain bitter taste perception. TAS2Rs belong to the GPCR family. Their activation follows the classical GPCR signal transduction pathway, converting the chemical bitter taste signal into an electrical signal, which is then transmitted to the nervous system (Figure 4).

When the bitter ligand binds to TAS2Rs, it induces conformational changes in the receptor, thereby activating its associated heterotrimeric G protein. In taste cells, the G protein is primarily Gα-gustducin. Receptor activation leads to the dissociation of the G protein into the Gα-gustducin subunit and the Gβγ dimer (typically composed of Gβ3 and Gγ13) [96,99]. The dissociated Gβγ subunit and Gα-gustducin subunit activate two parallel downstream signaling pathways, which together mediate the transduction and amplification of bitter taste signals.

The dissociated Gβγ subunits (particularly Gβ3 and Gγ13) are considered the primary mediators of bitter taste signaling, preferentially activating phospholipase Cβ2 (PLCβ2) [100,101]. Activated PLCβ2 catalyzes the hydrolysis of phosphatidylinositol-4, 5-diphosphate (PIP2) on the cell membrane, generating the key second messenger inositol 1,4,5-triphosphate (IP3) and diacylglycerol (DAG) [101]. IP3 binds to the IP3 receptor (IP3R) on the endoplasmic reticulum (ER), leading to the release of calcium ions (Ca^2+^) stored in the ER into the cytoplasm, thus significantly increasing intracellular Ca^2+^ levels [102]. Elevated intracellular Ca^2+^ is a crucial step in this pathway, activating transient receptor potential cation channel M member 5 (TRPM5) on the cell membrane [103]. Activation of TRPM5 results in the influx of sodium ions (Na^+^), leading to depolarization of the cell membrane [95]. Membrane depolarization further activates the calcium homeostasis modulator 1 (CALHM1) channel complex [104]. Activation of the CALHM1 channel promotes the release of adenosine triphosphate (ATP) as a neurotransmitter into the extracellular space. The released ATP activates ATP-gated ion channels (P2X2/3R) on adjacent afferent taste nerve fibers, ultimately transmitting bitter taste information to the central nervous system.

Compared to the Gβγ pathway, the details of the signaling pathway mediated by the Gα-gustducin subunit remain unclear. The known pathway involves activated Gα-gustducin activating phosphodiesterase (PDE), which subsequently degrades cyclic adenosine monophosphate (cAMP), leading to a reduction in intracellular cAMP levels. The reduction in cAMP may synergistically promote neurotransmitter release by relieving the inhibition of cyclic nucleotide-gated ion channels, allowing Ca^2+^ influx [105]. However, the specific contribution of this pathway to bitter taste perception and its downstream mechanisms require further clarification.

## 5. Bitter Flavor Masking and Removal Techniques

**Figure 5:**
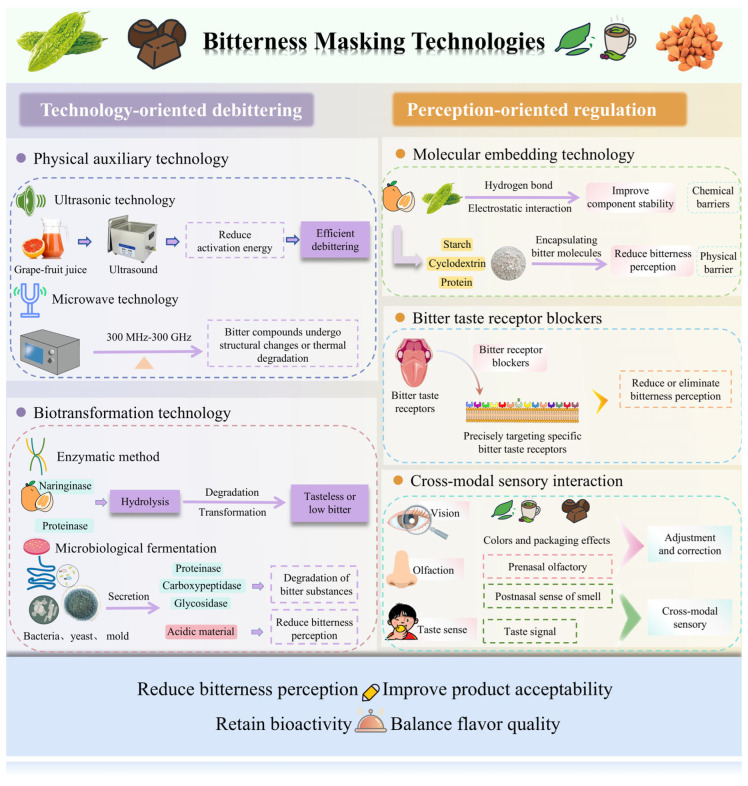
Different strategies to reduce the bitterness of foods.

Many bioactive compounds derived from MFPs and active pharmaceutical ingredients exhibit excellent health-promoting and therapeutic effects; however, their intense inherent bitterness and poor organoleptic properties severely limit the acceptability of MFPs and their derived products, as well as patient adherence. Therefore, effective intervention and masking of bitterness have transcended simple sensory modification, becoming a crucial link connecting the bioactivity of these compounds and their practical applicability in ethnopharmacological practice. Bitterness masking is not merely the removal of bitter molecules, but a series of technologies that reduce or modify unpleasant tastes by interfering with the entire pathway of bitter compounds—from their release in the herbal matrix, binding to taste receptors, to their final perception by the brain—through physical, chemical, or physiological approaches. (Figure 5, Table 3).

### 5.1. Technology-Driven De-Bittering Strategies

#### 5.1.1. Physically Assisted Techniques

Physical-assisted bitterness removal technology is a non-biological method that directly interacts with the MFPs matrix using specific physical energy fields (e.g., ultrasonic waves, microwaves, and thermal fields). It alters the molecular structure or state of bitter compounds through degradation, transformation, or volatilization, thereby reducing their sensory intensity. The key advantages of this technology are its high efficiency, environmental sustainability, and the absence of exogenous additives.

① Ultrasonic technology

Ultrasonic technology uses sound waves with frequencies exceeding the upper limit of human hearing (typically between 20 kHz and 10 MHz). Ultrasonic waves do not typically remove bitter compounds directly in the bitterness masking process. Instead, they assist in the de-bitterness process indirectly or alter the form of bitter substances through physical effects, such as mechanical, cavitation, and thermal effects. Specifically, ultrasonic treatment can alter the spatial conformation of enzymes and substrates, enhancing their affinity and significantly reducing thermodynamic energy barriers (e.g., activation energy, enthalpy change, and entropy change) in enzymatic reactions. This improves the enzymatic hydrolysis efficiency and rate of bitter precursors (e.g., proteins and polyphenols) [106]. Furthermore, ultrasound can mask bitterness through multiple pathways by degrading unstable bitter compounds or altering macromolecule structures (e.g., proteins), thus reducing their binding to taste bud receptors [107].

In the processing of *Citrus*-derived medicinal materials, such as *Citrus* grandis, ultrasound-assisted naringinase treatment (e.g., 50 kHz, 60 °C) significantly enhances the bitter effect [108]. The cavitation effect alters the enzyme and substrate (naringin) conformation, reducing the activation energy and increasing hydrolysis efficiency by approximately 20%, while also shortening processing time by nearly one-third. This approach achieves efficient debittering while maximally preserving the antioxidant constituents and bioactive phytochemicals of the *Citrus*, enabling a synergistic improvement in processing efficiency and product quality. In the de-bitterness treatment of *Lupinus albus* L., ultrasonic technology, through micro-jets and shock waves generated by the cavitation effect, effectively disrupts cell structure and accelerates the dissolution of water-soluble alkaloids [109]. Compared to the traditional water leaching method, this process reduces processing time by nearly 60%, demonstrating its potential as an efficient and eco-friendly physical bitterness removal method.

② Microwave technology

Microwave technology utilizes electromagnetic heating with frequencies ranging from 300 MHz to 300 GHz. The core principle is dielectric heating, where microwave energy causes polar molecules (mainly water molecules) in plant materials to rotate rapidly and collide, generating heat quickly [110]. This rapid and uniform heating causes structural changes or thermal degradation of heat-sensitive bitter compounds, thereby reducing their bitterness intensity. In a bitterness removal study of *Tinospora cordifolia* Miers., microwave treatment (100 W, 15 min) reduced the content of its main bitter substances (total terpenoids) by 26.5% [111]. Scanning electron microscopy results confirmed that microwaves caused cell rupture, providing a channel for the release of bitter compounds and subsequent thermal degradation. Additionally, this technology has the potential to optimize the extraction of other bioactive compounds (e.g., flavonoids) while eliminating bitterness. In autumn green tea processing, the microwave blanching process (500 W, 3 min) is used. It effectively retains chlorophyll, enhances the greenness and lightness of dry tea and tea soup, and significantly reduces the ratio of phenolic ammonia and ester-type catechins, thereby reducing bitterness and improving overall flavor [112].

Ultrasonic and microwave technologies, as representative physical-assisted bitterness removal methods, have provided key bitterness masking solutions for the MFPFs due to their high efficiency and sustainability. However, these technologies face challenges such as high equipment costs, precise energy control, and potential impacts on the overall quality of MFPs matrices when applied at scale. Future development should focus on synergizing multiple physical fields, precisely regulating process parameters, and innovatively combining these methods with other bitterness removal technologies to drive continuous progress.

#### 5.1.2. Bioconversion Technology

Biotransformation technology utilizes the specific catalytic activity of enzymes or microorganisms to directly degrade or transform bitter compounds, or to produce non-bitter and biologically beneficial derivatives. As such, it represents a highly efficient, selective and environmentally friendly strategy for bitterness regulation.

① Enzymatic de-bitterness method

The enzymatic bitterness removal method employs highly specific biological catalysts—enzymes—to convert bitter substances into tasteless or low-bitter compounds. This approach has been widely applied in the processing of citrus-derived medicinal materials and protein hydrolysates, relying on key enzymes such as naringinase and protease. Among these enzymes, naringinase plays a pivotal role. It is not a single enzyme but a complex system composed of α-L-rhamnosidase and β-D-glucosidase [113]. This enzyme system removes naringin (the primary bitter substance) through a two-step hydrolysis mechanism: First, α-L-rhamnosidase hydrolyzes the rhamnose bond at the naringin’s end, generating pullulan, which reduces bitterness intensity by about two-thirds. Next, β-D-glucosidase hydrolyzes pullulan into naringenin, which is entirely bitter-free, thereby eliminating the bitterness [114]. Protein bitterness primarily arises from hydrophobic bitter peptides produced during hydrolysis. A complex protease composed of endopeptidases and exopeptidases promotes the hydrolysis of specific peptide bonds in a coordinated manner, effectively reducing bitterness.

Although enzymatic methods have promising prospects, their practical application is often limited by issues such as short shelf life, poor stability, and sensitivity to process conditions. To address these issues, enzyme immobilization technology has become a focal point of research for achieving economically efficient continuous production [115]. When naringinase derived from Penicillium is immobilized on chitosan microspheres and applied to Citrus fruit juice, up to 75% of naringin can be removed. Immobilized enzymes exhibit superior stability compared to free enzymes in acidic fruit juice environments and can be reused, significantly reducing processing costs [114]. In olive debittering, the use of immobilized β-D-glucosidase can reduce oleuropein content by up to 80% within 6 h, much more efficiently than free enzymes (15.8%) and the traditional water-washing method [116]. The core advantage of the enzymatic method is its ability to precisely and efficiently “eliminate” specific bitter compounds. However, both free and immobilized enzymes function with the same focus: eliminating specific bitter compounds.

② Fermentation to remove bitterness

Microbial fermentation also serves as an effective debittering strategy. Through the metabolic activity of bacteria, yeasts and molds, bitter compounds can be degraded by secreted enzymes including proteases, carboxypeptidases and glycosidases. In addition, microbial metabolism can reduce bitterness perception by modulating the overall taste profile and degrading bitter secondary metabolites such as saponins and flavonoids. Lactic acid bacteria, in particular, have shown promising debittering effects via a dual mechanism: acid-induced taste masking and direct biodegradation of bitter constituents. Accordingly, microbial fermentation represents a mild, efficient and environmentally friendly approach for bitterness regulation in herbal materials.

Biotransformation technologies, particularly enzymatic and fermentation methods, provide effective natural solutions for masking bitterness. However, in practical applications, the activity of microorganisms is easily affected by environmental factors such as acidity, temperature, and salinity. Therefore, selecting suitable strains and precisely controlling fermentation conditions are essential to achieve stable and efficient bitterness removal.

### 5.2. Perception-Guided Regulation Pathway

The core of the perception-oriented regulatory pathway involves effectively masking bitterness by intervening in the taste perception process, exploiting sensory synergy effects, or modifying consumers’ cognitive expectations, without directly reducing or altering the bitter compounds. This can be summarized into three main strategies: First, constructing a physical barrier to prevent the release of bitter compounds in the oral cavity; second, using chemical agents to competitively block bitter taste receptors; and third, indirectly altering or masking bitterness perception through sensory stimulation from other flavor substances.

#### 5.2.1. Molecular Embedding Technology

Molecular embedding technology constructs physical or colloidal barriers using embedding agents such as cyclodextrin, starch, and proteins. These agents encapsulate bitter molecules, delay their release in the oral cavity, reduce contact with taste receptors, and thereby lower the perception of bitterness [117,118].

In traditional embedding materials, β-cyclodextrin effectively encapsulates hydrophobic bitter substances through host-guest interactions due to its cavity structure, which is hydrophilic on the outside and hydrophobic on the inside. Studies have shown that combining 0.7% β-cyclodextrin with ultrasound can reduce the levels of naringin and limonin in grapefruit peel by more than 50%, as well as reduce sensory bitterness by over 80% [119]. Modified starches exhibit varying release characteristics due to their structural differences. Porous starch, with its high specific surface area and strong adsorption capacity, achieves a slow and sustained release of caffeine. Its bitter taste-masking effect is superior to that of swelling starch and V-type starch [117]. Proteins primarily interact with bitter molecules through hydrogen bonds and hydrophobic interactions [120]. For instance, polymeric whey protein forms stable nanoparticles with notoginsenosides, effectively masking their bitterness [121].

The development of nanotechnology has further enhanced the precision of embedding systems. Nanocarriers, such as liposomes and solid lipid nanoparticles (SLNs), are widely used for the efficient encapsulation of bitter substances [122]. For instance, nano-liposomes encapsulating cucurbitacin mask its bitterness while retaining biological activity [123]. Chitosan-gelatin nanoparticles remain stable in a neutral oral environment and physically block the contact between triterpene saponins in red ginseng and taste receptors [124]. Furthermore, microencapsulation technology improves encapsulation efficiency and stability by constructing micron-sized wall materials [125]. For example, phospholipid nanocapsular microcapsules (PBN-M) encapsulate EGCG, and the microcapsule powder is prepared via spray drying to form a dual encapsulation system. This system achieves efficient flavor masking of polyphenols in aqueous phase systems [126].

However, at present, the research endpoints of the above-mentioned flavor masking systems are still mainly sensory evaluation and in vitro simulated release. The exploration of the stability, release kinetics and changes in oral bioavailability of the encapsulated active molecules in the complex gastrointestinal environment is relatively insufficient. Take β -cyclodextrin inclusion as an example. Although cyclodextrin can effectively mask the taste, it may also change the solubility and intestinal permeability of bitter molecules, thereby affecting its oral absorption process [127]; Similarly, although chitosan-gelatin nanoparticles effectively prevent triterpene saponins from coming into contact with taste buds under neutral oral conditions, the carriers may swelling or disintegrate when entering the acidic environment of the stomach, leading to premature release of active ingredients and a rebound of bitterness, ultimately altering the okinetic behavior of oral medications [124]. Therefore, the taste mask design of oral vectors must take into account the stability of gastric and intestinal fluids and drug release behavior. It is difficult to predict the pharmacokinetic characteristics in vivo based on in vitro data alone [128]; Future research urgently needs to systematically correlate taste masking performance with gastrointestinal release-absorption behavior.

Overall, the effectiveness of molecular embedding technology depends on the physicochemical properties of bitter substances and the choice of embedding materials. Although this technology has broad applicability, residual bitterness may still result from incomplete embedding. Therefore, in practical applications, it is necessary to systematically optimize the embedding process, gastrointestinal release behavior and the okinetic characteristics of oral drugs.

#### 5.2.2. Bitter Taste Receptor Blockers

The perception of bitterness begins with the activation of 25 human bitter taste receptors (hTAS2Rs) located on the tongue surface. These receptors are members of the GPCR family and can recognize hundreds of structurally diverse bitter compounds [19]. Bitter receptor blockers, or antagonists, are substances that specifically bind to these receptors, inhibiting their activation by bitter molecules. The core mechanism of action involves competitively occupying the orthosteric binding pocket of the receptor, preventing the binding of bitter agonists, and interrupting the perception of bitterness by blocking signal transduction.

Amino acid derivatives have shown significant potential among the discovered blockers. γ-Aminobutyric acid (GABA) and Nᵅ, Nᵋ-bis (carboxymethyl)-l-lysine (BCML) have been identified as highly effective blockers of the bitter taste receptor T2R4 [129]. These substances directly antagonize bitter taste signals produced by agonists, such as quinine, by competing for the same binding site. GABA is the first reported endogenous antagonist of T2R, while BCML exhibits strong inverse agonistic activity at nanomolar concentrations. Additionally, abscisic acid, a plant hormone, has been identified as a natural antagonist of hTAS2R4, effectively inhibiting receptor activation triggered by quinine [130].

The main advantage of the bitter taste receptor blocker strategy is its precision. It selectively targets and inhibits the activity of specific bitter taste receptors, thus achieving targeted masking of bitterness without affecting other basic tastes, such as sweetness and umami. However, this technology faces significant challenges. Humans have 25 subtypes of bitter taste receptors, and a single bitter substance can activate multiple receptors, forming a complex recognition network. Currently, the types of discovered blockers are limited, and none is universally applicable to all bitter taste receptors. Therefore, developing broad-spectrum or compound blocking strategies for complex bitter taste spectra is the key challenge and future research direction for efficient and comprehensive bitter taste removal.

#### 5.2.3. Cross-Modal Sensory Interaction

The overall flavor of MFPs results from the cross-modal integration of various sensory information in the brain, rather than being a simple combination of olfactory and gustatory signals. Aroma perception arises from volatile compounds stimulating olfactory receptors, including those in the prenasal and postnasal pathways. Taste perception, on the other hand, is triggered by non-volatile compounds activating taste receptors in the oral cavity. Although olfactory and gustatory signals have independent receptors and peripheral pathways, they interact significantly in the primary gustatory cortex and higher integration centers, such as the orbitofrontal cortex. In particular, the collaborative integration of aroma signals from postnasal olfaction and taste information plays a crucial role in modulating the sensory intensity of each signal [131]. This phenomenon is known as cross-modal sensory interaction.

The visual information of MFP products, such as their color and packaging, can significantly influence consumers’ taste perception by setting sensory expectations. Studies show that for bitter products, such as green tea, the greater the bitterness intensity, the more significant the impact of visual information on the final taste assessment. However, for products with milder bitterness, this effect is less pronounced [132]. In sensory perception, aroma plays a decisive role in modulating taste intensity. For bitter herbal medicines, pleasant volatile aromatic notes can divert sensory attention and interact cognitively with bitter signals, thereby softening the harshness of bitterness and improving the oral acceptability of herbal formulations [131].

In conclusion, the perceived intensity and palatability of bitterness in MFPs are largely regulated and corrected by olfactory and visual information. The aroma behind the nose can directly suppress the bitter taste signal through central integration, while visual cues modulate the subsequent taste experience by presetting sensory expectations. These cross-modal methods offer multi-dimensional, innovative strategies for managing bitterness. However, the precise mechanisms of sensory interaction are complex, with significant individual differences. In the future, systematic sensory evaluation and consumer research will be necessary to identify the optimal sensory balance for specific products.

**Table 3:** Different Methods for Reducing Bitterness.

Method Category		Advantages	Disadvantages	Application	References
Physical Assistive Technology	Ultrasonic Technology	Highly efficient, energy-saving, environmentally friendly; improves processing efficiency and sterilization; degrades unstable bitter compounds.	Indirect/non-specific; may affect other functional compounds.	Debittering citrus peel.	[10]
	Microwave Technology	Thermally degrades bitter compounds; enhances permeability.	High equipment cost; risk of local overheating or cavitation damage.	Reducing bitterness in green tea.	[112]
Bioconversion Technology	Enzymatic	High efficiency and specificity; mild conditions; directly degrades bitter peptides/glycosides; preserves nutrition.	High cost and instability of enzymes; requires precise control; may generate new bitter compounds.	Debittering citrus-derived herbal materials.	[113]
	Microbial Fermentation	Natural and multifunctional (enhances nutrition, flavor, shelf-life); degrades various bitter compounds; produces masking flavors and organic acids.	Sensitive to environmental factors; requires careful strain selection and process control.	Lactic acid bacteria fermentation reduces bitterness in medicinal and edible plant extracts.	[133]
Perception-Guided Regulation Pathway	Molecular embedding	Effectively isolates and protects active ingredients; multifunctional.	Material/process selection is compound-specific; some high-end options are costly.	Microencapsulation of *Panax notoginseng* saponins to reduce bitterness.	[134]
	Bitter taste receptor antagonists	High specificity and low dosage; retains original bitter compounds.	High cost; potential safety concerns.	Abscisic acid inhibits the hTAS2R4 receptor.	[130]
	Cross-modal sensory interaction	Enhances acceptability by improving overall sensory experience.	Does not directly remove bitterness; may alter product flavor profile.	Pleasant volatile aroma from herbal materials can mellow bitterness in herbal preparations.	[135]

## 6. Summary and Outlook

Bitterness holds a crucial position in the “Five Flavors” system of traditional Chinese medicine. It not only carries the traditional understanding of the properties of “draining, drying and strengthening”, but also substantially governs the sensory acceptance and clinical compliance of plants that are both food and medicine. This article follows the main line of “structure-perception-regulation”, systematically sorts out the natural sources, chemical types, separation and identification methods, sensory and receptor-mediated perception mechanisms of bitter compounds, and reviews the strategic evolution from traditional taste correction ideas to contemporary taste masking and bitterness removal technologies.

At the material basis level, compounds such as alkaloids, terpenoids, polyphenols and bitter glycosides not only endow medicinal food plants with diverse biological activities, but also constitute the main source of the bitter taste. Classic methods such as traditional sensory-guided separation and chromatography-mass spectrometry have formed a complementary pattern with high-throughput methods such as virtual screening and metabolomics, significantly improving the detection efficiency of bitter components in complex matrices. In terms of sensory evaluation, a well-trained tasting team, along with objective instruments such as electronic tongues, jointly form the hub for determining the intensity and quality of bitterness. To address the palatability challenge, a series of technical strategies has been established: direct conversion or removal of bitter molecules through physical and biological degradation methods; molecular inclusion, receptor antagonism and cross-modal interaction regulation finely modulated from the sensory perspective. Behind these strategies lies an academic background worth noting-the study of traditional Chinese medicine formulations has long been using the compatibility and restraint of medicinal flavors in practice to balance the bitter taste. This experience-level awareness of taste regulation provides a simple yet profound ideological guide for today’s molecular-level taste correction research. This article also systematically summarizes the molecular mechanisms of bitter taste signal transduction, especially the extensive expression of the TAS2Rs family in oral and extra-oral tissues such as the intestine and respiratory tract, suggesting a deep connection between bitter taste perception and the functions of metabolic, immune and other systems. However, the causal relationship and specific regulatory pathways between the bitter taste signal mediated by external TAS2Rs and metabolism, immunity, and systemic physiological functions are currently mostly emerging hypotheses based on preliminary experimental evidence, and there is still a lack of direct support from large-scale human studies and clinical data. Overall, however, current research mostly focuses on a single link in the “structure-perception-regulation” chain. There is significant heterogeneity in methodological standards among different laboratories for the identification of bitter components, sensory intensity evaluation, and verification of regulatory effects. A standardized research paradigm that spans the entire chain has not yet been formed. This also limits the large-scale application and transformation of the framework in the fields of pharmacology and food science.

Although significant progress has been made, this field still faces multiple challenges. Firstly, the complete structure, flavor threshold and in vivo metabolic pathways of a large number of bitter compounds remain unclear, and the synergistic and antagonistic effects among coexisting compounds urgently need systematic research. Secondly, when dealing with highly polar, low-abundance or unstable bitter substances, the separation and identification techniques still need to further enhance selectivity and throughput. Targeted enrichment methods such as affinity ultrafiltration and cell membrane chromatography offer feasible research ideas. Thirdly, sensory evaluation is gradually shifting from reliance on manual tasting to the integration of instruments and neuroscience. Although central nervous system measurement methods such as electroencephalogram (EEG) and functional near-infrared spectroscopy (fNIRS) have provided new directions for the objective quantification of bitter taste perception, these methods still have obvious limitations in the field of taste research at present: The lack of unified norms for experimental paradigms and data processing procedures leads to significant differences in individual neural responses, resulting in insufficient reproducibility of the results. Moreover, there are still technical bottlenecks in the precise delivery of taste stimuli and the traceability and analysis of neural signals. In practical applications, it is mostly limited to the mechanism exploration of small samples and has not yet been extended to the sensory evaluation of large-scale samples. In the future, on the one hand, the sensitivity and selectivity of the electronic tongue should be continuously improved. On the other hand, the experimental process of neuroscientific methods should be gradually standardized, and stable quantitative indicators of the bitter taste central response should be established to promote its transformation from basic research to practical evaluation tools.

In future research, green sustainability should be the priority principle of bitterness removal technology. At the same time, it is necessary to deeply clarify the interaction mode between bitter compounds and TAS2Rs, reveal the molecular basis of “taste and efficacy correlation”, and provide new tools for the quality evaluation of traditional Chinese medicine. Methodological standardization oriented towards the “structure-perception-regulation” framework can be advanced in two scenarios: In terms of the evidence base, for non-targeted metabolomics data, it is necessary to implement the standard for the classification and reporting of metabolite identification confidence levels. The sensory evaluation team should unify the screening and training standards for evaluators and the threshold determination paradigm. For bioinformatics virtual screening, the selection basis of the docking algorithm, scoring function and positive determination threshold should be clearly defined to reduce the conclusion bias caused by methodological heterogeneity among different studies. On this basis, a standardized research process of “structural analysis-receptor verification-pharmacodynamic association” can be established in the field of pharmacology. Bitter taste perception can be used as an alternative marker for the activity screening and quality evaluation of traditional Chinese medicine, and the operation norms and interpretation standards can be unified. In the field of food science, a standardized decision-making path of “bitterness recognition-sensory characterization-technical adaptation” can be established. For different matrices, a grading screening scheme for flavor masking technology can be formed, providing a reusable paradigm for product development. This review aims to promote the development of more efficient and safe bitter taste regulation strategies through interdisciplinary integration, facilitate the modernization of bitter-tasting traditional Chinese medicine, and promote the continuous value of traditional food and medicine resources in the modern health system.

## Data Availability

All data generated or analyzed during this study are included in this published article.
